# Supplementary material for: Enteric pharmacokinetics of monomeric and multimeric camelid nanobody single-domain antibodies
Source: PLoS One. 2023 Nov 27;18(11):e0291937. doi: 10.1371/journal.pone.0291937 (PMC10681176; doi:10.1371/journal.pone.0291937)
Supplement: S1 Fig — A 20 μl aliquot of each sample from Fig 2B was incubated with 50 ng of LC/A and SNAP25 substrate (BoTest™ A/E, BioSentinel Pharmaceuticals) [1] in a 50 μl reaction. Half of each reaction was quenched after either 15 min or 60 min incubation at 37°C and quenched by boiling in 1x SDS sample buffer. SDS PAGE was performed on 10 μl aliquots, transferred to filters and probed with 1:1000 rabbit HRP/anti-GFP (Santa Cruz). Western blots are shown on the samples incubated for 15 min with LC/A (top) or 60 min with LC/A (bottom). In addition, a control lane is included (No ALc, 60m) in which the SNAP25 was incubated for 60 min in PBS without LC/A. The relative amount of uncleaved SNAP25 substrate was assessed using an imaging system by comparing the signal to that of undigested substrate (0m, PBS). The data in Fig 2C reflects the quantified signal of the full-size SNAP25 in each lane as a % of the ‘PBS 0m’ control signal. Samples able to neutralize LC/A inhibition after chyme incubations protect the substrate from LC/A digestion, thus resulting in uncleaved SNAP25. The lower bands represent unquantified SNAP25 degradation products. Note, some of these degradation products likely derive from residual chyme protease activity not fully inhibited by the chyme incubation termination quench. (PDF) [file pone.0291937.s001.pdf]

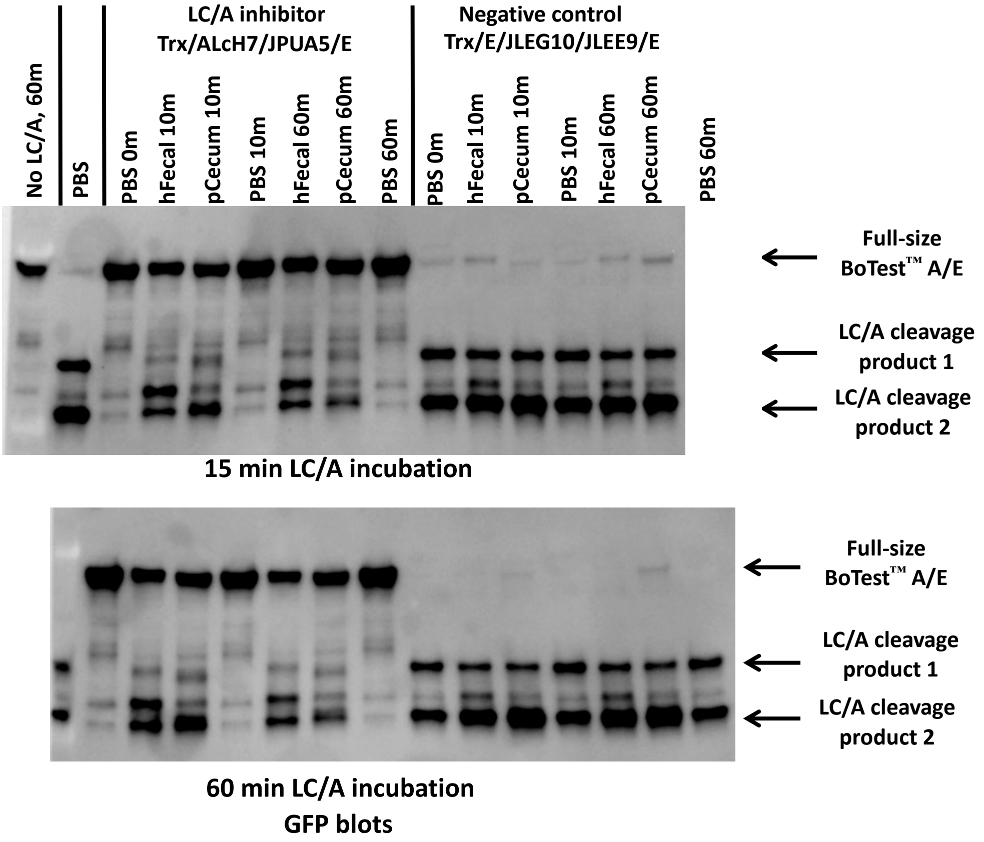

S1 Fig. LC/A inhibition assay data in support of Fig 2. A 20 µl aliquot of each sample from Fig 2B was incubated with 50 ng of LC/A and SNAP25 substrate (BoTest™ A/E, BioSentinel Pharmaceuticals) [1] in a 50 µl reaction. Half of each reaction was quenched after either 15 min or 60 min incubation at 37 °C and quenched by boiling in 1x SDS sample buffer. SDS PAGE was performed on 10 µl aliquots, transferred to filters and probed with 1:1000 rabbit HRP/anti-GFP (Santa Cruz). Western blots are shown on the samples incubated for 15 min with LC/A (top) or 60 min with LC/A (bottom). In addition, a control lane is included (No ALc, 60m) in which the SNAP25 was incubated for 60 min in PBS without LC/A. The relative amount of uncleaved SNAP25 substrate was assessed using an imaging system by comparing the signal to that of undigested substrate (0m, PBS). The data in Fig. 2C reflects the quantified signal of the full-size SNAP25 in each lane as a % of the 'PBS 0m' control signal. Samples able to neutralize LC/A inhibition after chyme incubations protect the substrate from LC/A digestion, thus resulting in uncleaved SNAP25. The lower bands represent unquantified SNAP25 degradation products. Note, some of these degradation products likely derive from residual chyme protease activity not fully inhibited by the chyme incubation termination quench.

References

1. Ruge DR, Dunning FM, Piazza TM, Molles BE, Adler M, Zeytin FN, et al. Detection of six serotypes of botulinum neurotoxin using fluorogenic reporters. Anal Biochem. 2011;411(2):200-9. Epub 2011/01/11. doi: 10.1016/j.ab.2011.01.002. PubMed PMID: 21216216.
