# Supplementary material for: Enteric pharmacokinetics of monomeric and multimeric camelid nanobody single-domain antibodies
Source: PLoS One. 2023 Nov 27;18(11):e0291937. doi: 10.1371/journal.pone.0291937 (PMC10681176; doi:10.1371/journal.pone.0291937)
Supplement: S2 Fig — (A) Coomassie-stained SDS-PAGE (5 μg of VHH agent/lane) and (B) Coomassie-stained PVDF membrane obtained following transfer of the same samples from a preparative SDS-PAGE gel (10 μg of VHH agent/lane). All samples derive from chyme incubations of VHH heterodimer Trx/E/AH3/AA6/E (500 μg/ml) [2] (diagram shown in C). This VHH agent was incubated for 60 min (60m) or overnight (o/n) with 1:30 pig intestinal extract or 1:30 human fecal extract before SDS-PAGE. Untreated VHH (0) was utilized as a control. The two ∼16 kDa digestion products indicated in B with an arrow were submitted for amino terminal Edman sequence analysis (S3A and S3B Fig). (PDF) [file pone.0291937.s002.pdf]

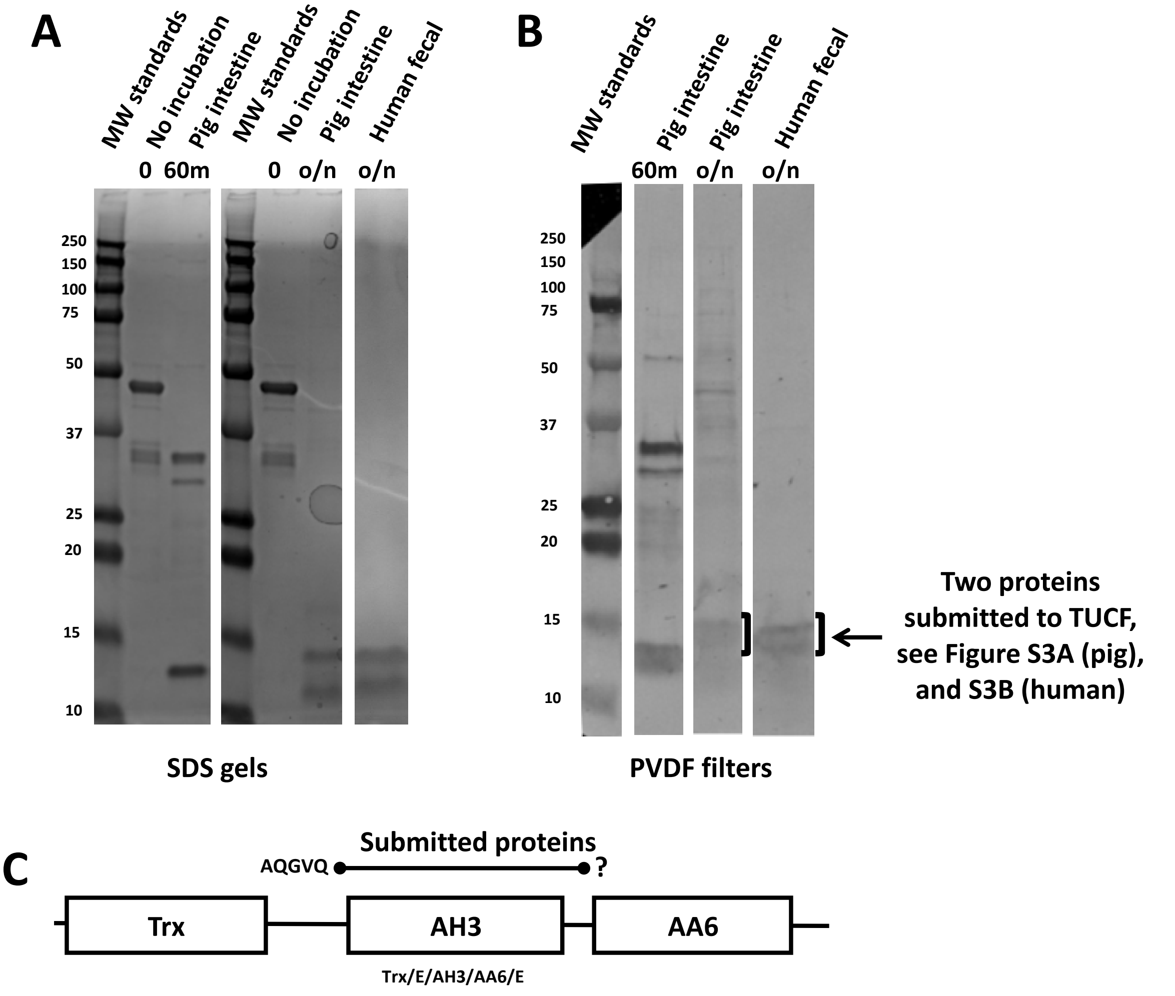

S2 Fig. Edman amino terminal amino acid sequences of the VHH heterodimer Trx/E/AH3/AA6/E in support of Fig 3. (A) Coomassie-stained SDS-PAGE (5 µg of VHH agent/lane) and **(B)** Coomassie-stained PVDF membrane obtained following transfer of the same samples from a preparative SDS-PAGE gel (10 µg of VHH agent/lane). All samples derive from chyme incubations of VHH heterodimer Trx/E/AH3/AA6/E (500 µg/ml) [2] (diagram shown in **C**). This VHH agent was incubated for 60 min (60m) or overnight (o/n) with 1:30 pig intestinal extract or 1:30 human fecal extract before SDS-PAGE. Untreated VHH (0) was utilized as a control. The two ~16 kDa digestion products indicated in B with an arrow were submitted for amino terminal Edman sequence analysis (**S3A and S3B Figs**).

References

2. Yang Z, Schmidt D, Liu W, Li S, Shi L, Sheng J, et al. A novel multivalent, single-domain antibody targeting TcdA and TcdB prevents fulminant Clostridium difficile infection in mice. J Infect Dis. 2014. doi: 10.1093/infdis/jiu196. PubMed PMID: 24683195.
