# Supplementary material for: Enteric pharmacokinetics of monomeric and multimeric camelid nanobody single-domain antibodies
Source: PLoS One. 2023 Nov 27;18(11):e0291937. doi: 10.1371/journal.pone.0291937 (PMC10681176; doi:10.1371/journal.pone.0291937)
Supplement: S3 Fig — Edman degradation traces of VHH heterodimer Trx/E/AH3/AA6/E digestion products from S2 Fig resulting from incubation with (A) porcine intestinal chyme or (B) human fecal extract. The five traces corresponding to the first five residues are shown with the top candidates for amino acids 1 to 5 indicated with arrows. The top amino acid calls (identified blind) are shown to the right, with secondary calls in parentheses. The sequencing results of both bands indicated an amino terminus of AQGVQ which could be unambiguously identified at the amino end of the submitted peptide shown in Figs 3 and S2C. (PDF) [file pone.0291937.s003.pdf]

A

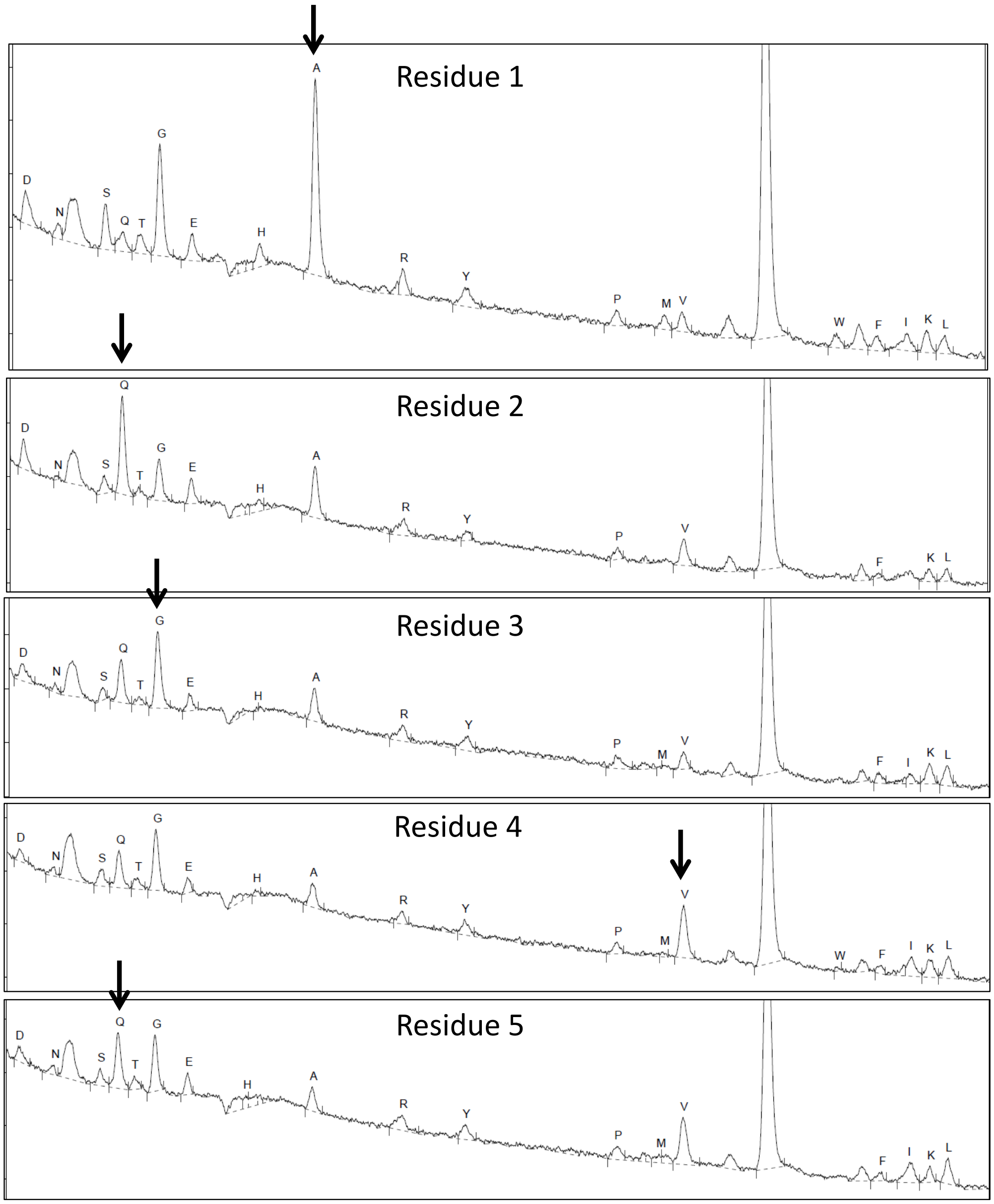

Amino acid calls by TUCF:

1. **A, (S, H)**
2. **Q, (G, V, L, F, Y)**
3. **G, (L, I)**
4. **V, (T)**
5. **Q, (L)**

B

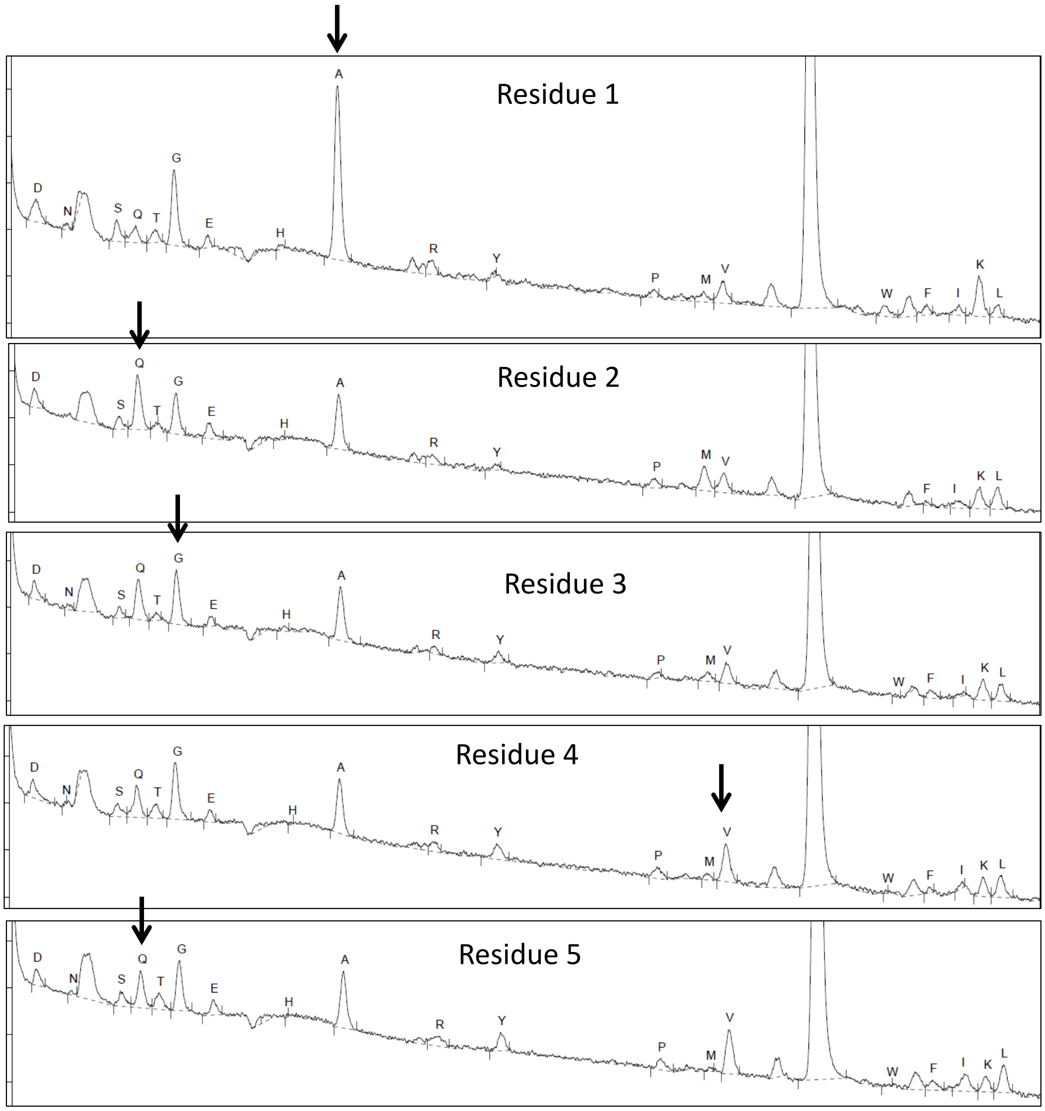

Amino acid calls by TUCF:

1. **A**
2. **Q, (M, L)**
3. **G**
4. **V**
5. **Q, (Y, V)**

S3 Fig. Amino acid analysis traces for VHH heterodimer Trx/E/AH3/AA6/E sequential Edman degradation of sample from S2 Fig. Edman degradation traces of VHH heterodimer Trx/E/AH3/AA6/E digestion products from **S2 Fig** resulting from incubation with **(A)** porcine intestinal chyme or **(B)** human fecal extract. The five traces corresponding to the first five residues are shown with the top candidates for amino acids 1 to 5 indicated with arrows. The top amino acid calls (identified blind) are shown to the right, with secondary calls in parentheses. The sequencing results of both bands indicated an amino terminus of AQGVQ which could be unambiguously identified at the amino end of the submitted peptide shown in **Fig 3** and **S2C Figs**.
