# Supplementary material for: Enteric pharmacokinetics of monomeric and multimeric camelid nanobody single-domain antibodies
Source: PLoS One. 2023 Nov 27;18(11):e0291937. doi: 10.1371/journal.pone.0291937 (PMC10681176; doi:10.1371/journal.pone.0291937)
Supplement: S4 Fig — (A) Coomassie-stained SDS- PAGE (2 μg/lane) and (B) Coomassie-stained PVDF membrane obtained following transfer of the same samples from a preparative SDS-PAGE gel (4 μg of VHH agent/lane). Samples derive from chyme incubation of VHH heterodimer Trx/E/JDQF12/JDQD12/E (40 μg/ml) [3] (diagram shown in C). This VHH agent was incubated for 10 min (10m) or 60 min (60m) with 1:10 pig intestinal extract before samples were loaded to each lane. Untreated VHH (0) was included as a control. The ∼30 kDa digestion product indicated in B was submitted for amino terminal Edman sequence analysis (S5 Fig). (PDF) [file pone.0291937.s004.pdf]

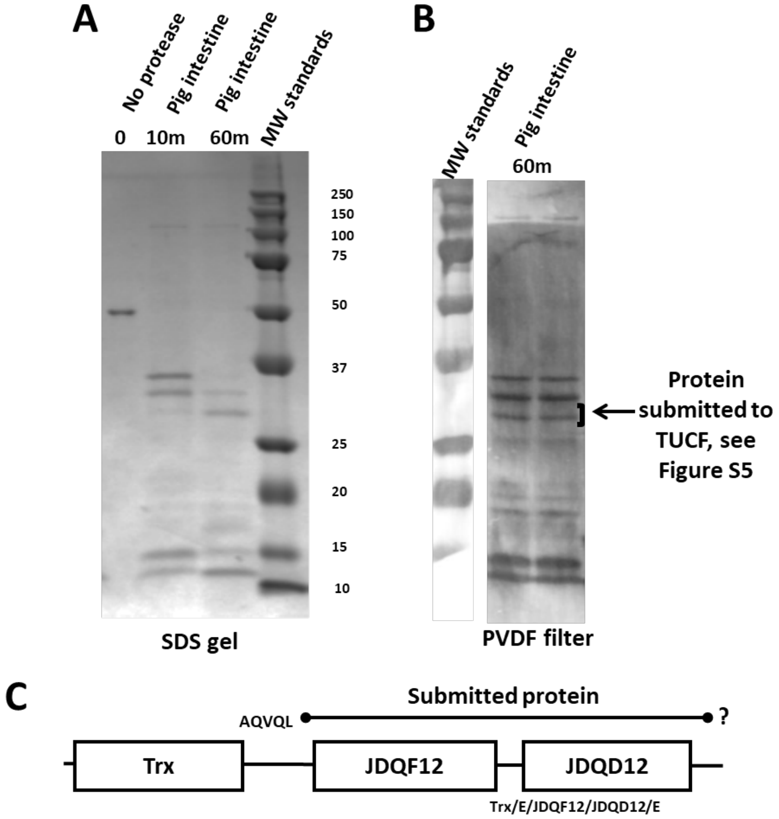

S4 Fig. Edman amino terminal amino acid sequence of the VHH heterodimer Trx/E/JDQF12/JDQD12/E in support of Fig 3. (A) Coomassie-stained SDS- PAGE (2 μg/lane) and **(B)** Coomassie-stained PVDF membrane obtained following transfer of the same samples from a preparative SDS-PAGE gel (4 μg of VHH agent/lane). Samples derive from chyme incubation of VHH heterodimer Trx/E/JDQF12/JDQD12/E (40 μg/ml) [3] (diagram shown in **C**). This VHH agent was incubated for 10 min (10m) or 60 min (60m) with 1:10 pig intestinal extract before samples were loaded to each lane. Untreated VHH (0) was included as a control. The ~30 kDa digestion product indicated in B was submitted for amino terminal Edman sequence analysis (**S5 Fig**).

References

3. Mukherjee J, Tremblay JM, Leysath CE, Ofori K, Baldwin K, Feng X, et al. A novel strategy for development of recombinant antitoxin therapeutics tested in a mouse botulism model. PLoS One. 2012;7(1):e29941. Epub 2012/01/13. doi: 10.1371/journal.pone.0029941. PubMed PMID: 22238680; PubMed Central PMCID: PMCPMC3253120.
