# Supplementary material for: Enteric pharmacokinetics of monomeric and multimeric camelid nanobody single-domain antibodies
Source: PLoS One. 2023 Nov 27;18(11):e0291937. doi: 10.1371/journal.pone.0291937 (PMC10681176; doi:10.1371/journal.pone.0291937)
Supplement: S6 Fig — (A) Coomassie-stained PVDF membrane of samples that derive from chyme incubation of VHH heterodimer 6H/E/StxA5/Stx2G1/E [4] (diagram shown in B). This VHH agent (500 μg/ml) was incubated with pig intestinal extract (1:30) and 2.5 μg VHH heteromultimer was then loaded per lane. After 60 min, this ∼33 kDa VHH heterodimer resulted in a single digestion product of ∼14 kDa that likely consisted of both monomer VHH components. The band was excised as indicated in A and submitted for amino terminal Edman sequence analysis (S7 Fig). (PDF) [file pone.0291937.s006.pdf]

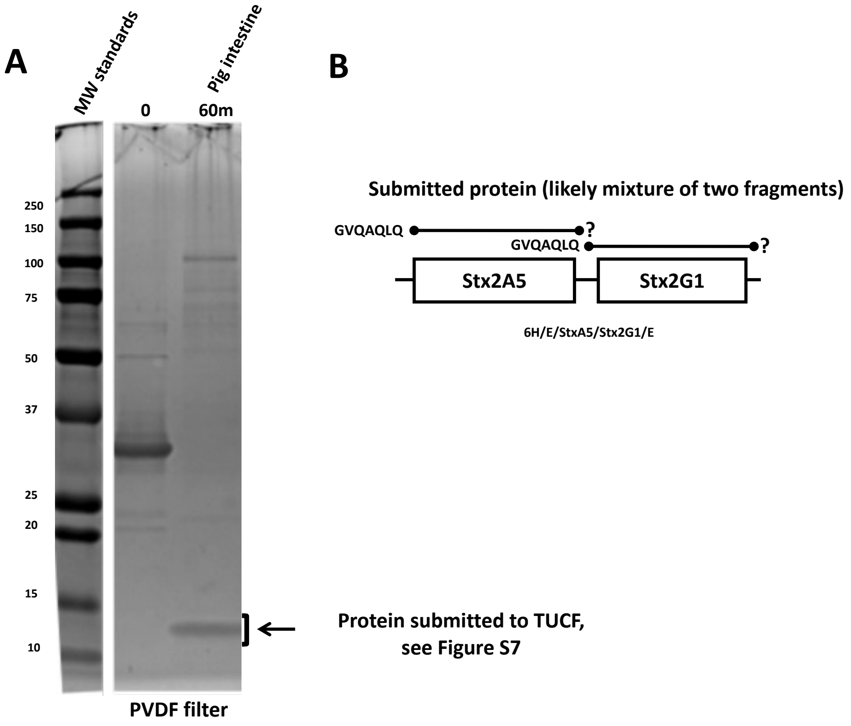

S6 Fig. Edman amino terminal amino acid sequence of the VHH heterodimer 6H/E/StxA5/Stx2G1/E in support of Fig 3. (A) Coomassie-stained PVDF membrane of samples that derive from chyme incubation of VHH heterodimer 6H/E/StxA5/Stx2G1/E [4] (diagram shown in **B**). This VHH agent (500 µg/ml) was incubated with pig intestinal extract (1:30) and 2.5 µg VHH heteromultimer was then loaded per lane. After 60 min, this ~33 kDa VHH heterodimer resulted in a single digestion product of ~14 kDa that likely consisted of both monomer VHH components. The band was excised as indicated in **A** and submitted for amino terminal Edman sequence analysis (**S7 Fig**).

References

4. Tremblay JM, Mukherjee J, Leysath CE, Debatis M, Ofori K, Baldwin K, et al. A single VHH-based toxin-neutralizing agent and an effector antibody protect mice against challenge with Shiga toxins 1 and 2. Infection and immunity. 2013;81(12):4592-603. doi: 10.1128/IAI.01033-13. PubMed PMID: 24082082; PubMed Central PMCID: PMC3837998.
