# Supplementary material for: Enteric pharmacokinetics of monomeric and multimeric camelid nanobody single-domain antibodies
Source: PLoS One. 2023 Nov 27;18(11):e0291937. doi: 10.1371/journal.pone.0291937 (PMC10681176; doi:10.1371/journal.pone.0291937)
Supplement: S7 Fig — Edman degradation traces of VHH heterodimer 6H/E/StxA5/Stx2G1/E digestion products from S6 Fig. The seven traces corresponding to the first seven residues are shown with the top candidates for amino acids 1 to 7 indicated with arrows. The top amino acid calls (identified blind) are shown at the top, with secondary calls in parentheses. We conclude that the correct amino end call for residue 1 must be a glycine as no similar sequence existed in this protein in which alanine could be the correct call. The sequencing results of the degradation product(s) indicated an amino terminus of GVQAQLQ, a sequence found immediately upstream of both core VHHs as shown in S6B Fig. We suggest that the submitted product was most likely a pool of two degradation products resulting from cleavages at these two sites in both VHHs. We cannot rule out that the band resulted from cleavage at just one of the two identified sites while the second VHH was further degraded to smaller fragments. (PDF) [file pone.0291937.s007.pdf]

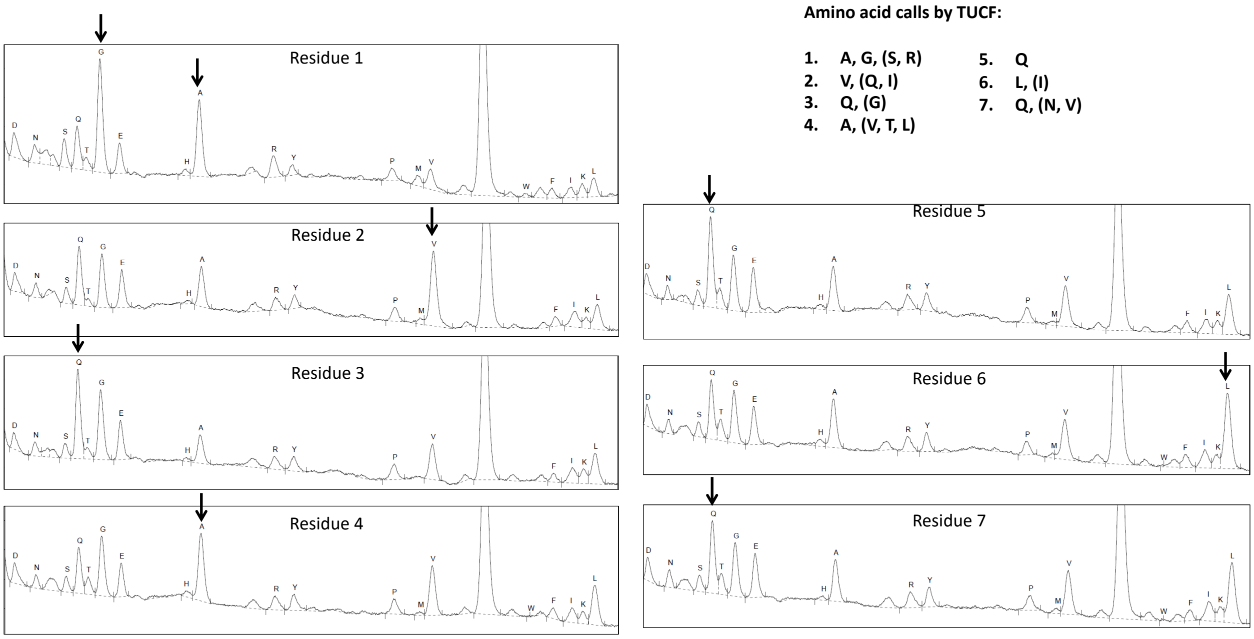

S7 Fig. Amino acid analysis traces for VHH heterodimer 6H/E/StxA5/Stx2G1/E sequential Edman degradation of sample from S6 Fig. Edman degradation traces of VHH heterodimer 6H/E/StxA5/Stx2G1/E digestion products from **S6 Fig**. The seven traces corresponding to the first seven residues are shown with the top candidates for amino acids 1 to 7 indicated with arrows. The top amino acid calls (identified blind) are shown at the top, with secondary calls in parentheses. We conclude that the correct amino end call for residue 1 must be a glycine as no similar sequence existed in this protein in which alanine could be the correct call. The sequencing results of the degradation product(s) indicated an amino terminus of GVQAQLQ, a sequence found immediately upstream of both core VHHs as shown in **S6B Fig**. We suggest that the submitted product was most likely a pool of two degradation products resulting from cleavages at these two sites in both VHHs. We cannot rule out that the band resulted from cleavage at just one of the two identified sites while the second VHH was further degraded to smaller fragments.
