# Supplementary material for: Enteric pharmacokinetics of monomeric and multimeric camelid nanobody single-domain antibodies
Source: PLoS One. 2023 Nov 27;18(11):e0291937. doi: 10.1371/journal.pone.0291937 (PMC10681176; doi:10.1371/journal.pone.0291937)
Supplement: S8 Fig — (A) Cartoons of the three VHH heterodimer proteins tested. Each heterodimer was identical except for the sequence of spacer region as indicated in S2 Table. Each consisted of two Stx2-neutralizing core VHHs, JGH-G1 (G1) and JFG-H6 (H6) [4], an amino end hexahistidine for purification and a carboxyl end myc tag. 6H/G1/5G/H6 had a spacer consisting of 5 glycine residues (5G), 6H/G1/PG3/H6 had a spacer consisting of the six amino acid sequence PGPGPG (PG3), and 6H/G1/PE3/H6 had a spacer consisting of the six amino acid sequence PEPEPE (PE3). (B) The three VHH heterodimers (50 μg/ml) were each subjected to incubations with rabbit chyme (1:10) for the indicated times and quenched on ice with 1x HALT protease inhibitor cocktail. The resulting samples were diluted 1:1 with 2x SDS sample buffer and were then assayed for their integrity by western blot (10 μl loaded per lane) probed with rabbit anti-denatured VHH sera. (PDF) [file pone.0291937.s008.pdf]

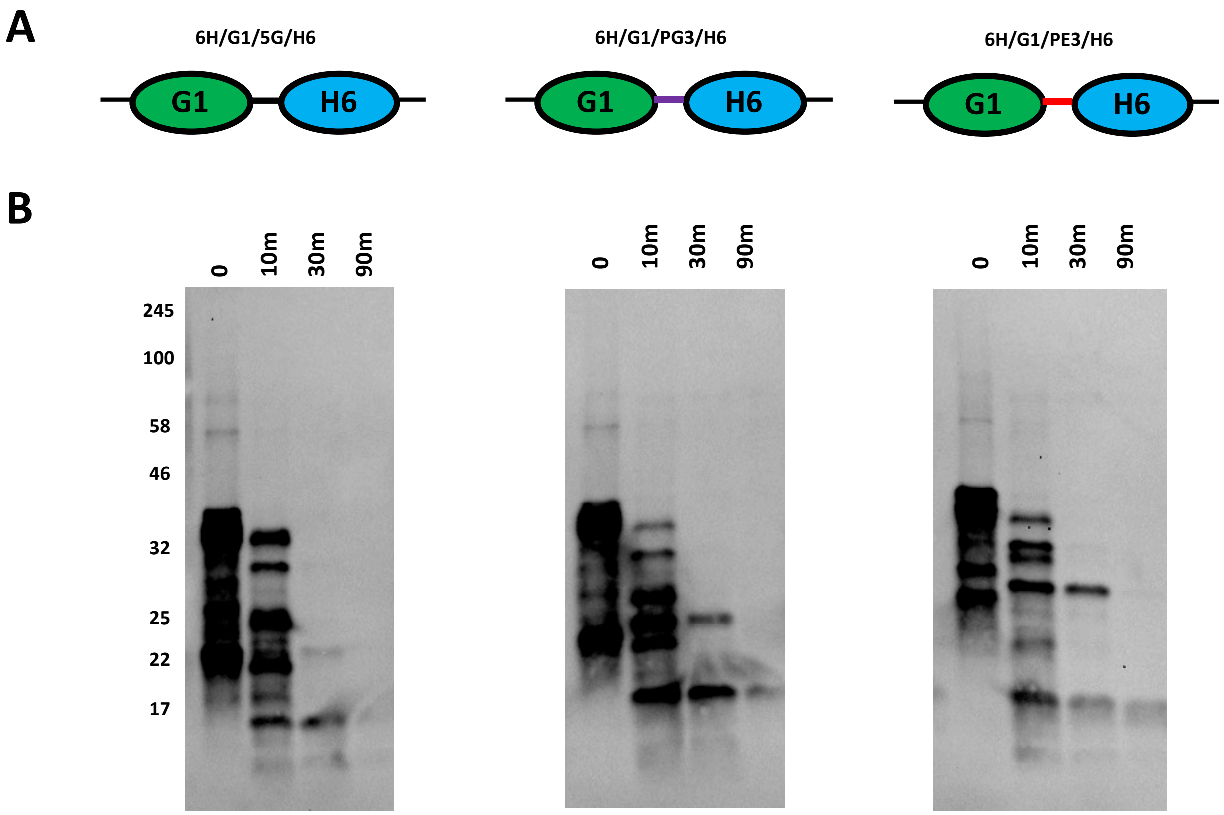

S8 Fig. Enteric proteases similarly cleave a VHH dimer varying in spacer sequences. **(A)** Cartoons of the three VHH heterodimer proteins tested. Each heterodimer was identical except for the sequence of spacer region as indicated in **S2 Table**. Each consisted of two Stx2-neutralizing core VHHs, JGH-G1 (G1) and JFG-H6 (H6) [4], an amino end hexahistidine for purification and a carboxyl end myc tag. 6H/G1/5G/H6 had a spacer consisting of 5 glycine residues (5G), 6H/G1/PG3/H6 had a spacer consisting of the six amino acid sequence PGPGPG (PG3), and 6H/G1/PE3/H6 had a spacer consisting of the six amino acid sequence PEPEPE (PE3). **(B)** The three VHH heterodimers (50 µg/ml) were each subjected to incubations with rabbit chyme (1:10) for the indicated times and quenched on ice with 1x HALT protease inhibitor cocktail. The resulting samples were diluted 1:1 with 2x SDS sample buffer and were then assayed for their integrity by western blot (10 µl loaded per lane) probed with rabbit anti-denatured VHH sera.

References

4. Tremblay JM, Mukherjee J, Leysath CE, Debatis M, Ofori K, Baldwin K, et al. A single VHH-based toxin-neutralizing agent and an effector antibody protect mice against challenge with Shiga toxins 1 and 2. Infection and immunity. 2013;81(12):4592-603. doi: 10.1128/IAI.01033-13. PubMed PMID: 24082082; PubMed Central PMCID: PMC3837998.
