# Supplementary material for: Enteric pharmacokinetics of monomeric and multimeric camelid nanobody single-domain antibodies
Source: PLoS One. 2023 Nov 27;18(11):e0291937. doi: 10.1371/journal.pone.0291937 (PMC10681176; doi:10.1371/journal.pone.0291937)
Supplement: S1 Table — Colored sequences correspond to VHH components diagramed in Fig 7A. (PDF) [file pone.0291937.s009.pdf]

S1 Table. Flanking and spacer sequences of TcdB-neutralizing VHH heterodimer from Fig. 7. Colored sequences correspond to VHH components diagramed in **Fig. 7A**.

NAME	VHH HETERODIMER STRUCTURE	RELEVANT AMINO ACID SEQUENCE
6H/5D/0/E3	No spacer, standard flanking region	QVQLVE...VHH1....VSS/QVQLVE...VHH2....VSS
6H/PE5/5D/0/E3/PE5	No spacer, flanking proline-rich region	PEPEPEPEPE/QVQLVE...VHH1....VSS/QVQLVE...VHH2....VSS/PEPEPEPEPE
6H/PE5/5D/15G/E3/PE5	PolyG spacer, flanking proline-rich region	PEPEPEPEPE/QVQLVE...VHH1....VSS/GGGGGGGGGGGGGGG/QVQLVE...VHH2....VSS/PEPEPEPEPE
