# Supplementary material for: Enteric pharmacokinetics of monomeric and multimeric camelid nanobody single-domain antibodies
Source: PLoS One. 2023 Nov 27;18(11):e0291937. doi: 10.1371/journal.pone.0291937 (PMC10681176; doi:10.1371/journal.pone.0291937)
Supplement: S2 Table — Colored sequences correspond to VHH components diagramed in S8A Fig. (PDF) [file pone.0291937.s010.pdf]

S2 Table. Flanking and spacer sequences of two Stx2-neutralizing VHH heterodimer from S8 Fig. Colored sequences correspond to VHH components diagramed in **S8A Fig**.

NAME	SPACER	RELEVANT AMINO ACID SEQUENCE
6H/G1/5G/H6	GGGGG	QVQLVE...VHH1....VSS/GGGGG/QVQLVE...VHH2....VSS
6H/G1/PG3/H6	PGPGPG	QVQLVE...VHH1....VSS/PGPGPG/QVQLVE...VHH2....VSS
6H/G1/PE3/H6	PEPEPE	QVQLVE...VHH1....VSS/PEPEPE/QVQLVE...VHH2....VSS
