# Supplementary material for: Enteric pharmacokinetics of monomeric and multimeric camelid nanobody single-domain antibodies
Source: PLoS One. 2023 Nov 27;18(11):e0291937. doi: 10.1371/journal.pone.0291937 (PMC10681176; doi:10.1371/journal.pone.0291937)
Supplement: S1 Raw images — (PDF) [file pone.0291937.s012.pdf]

Fig. 1

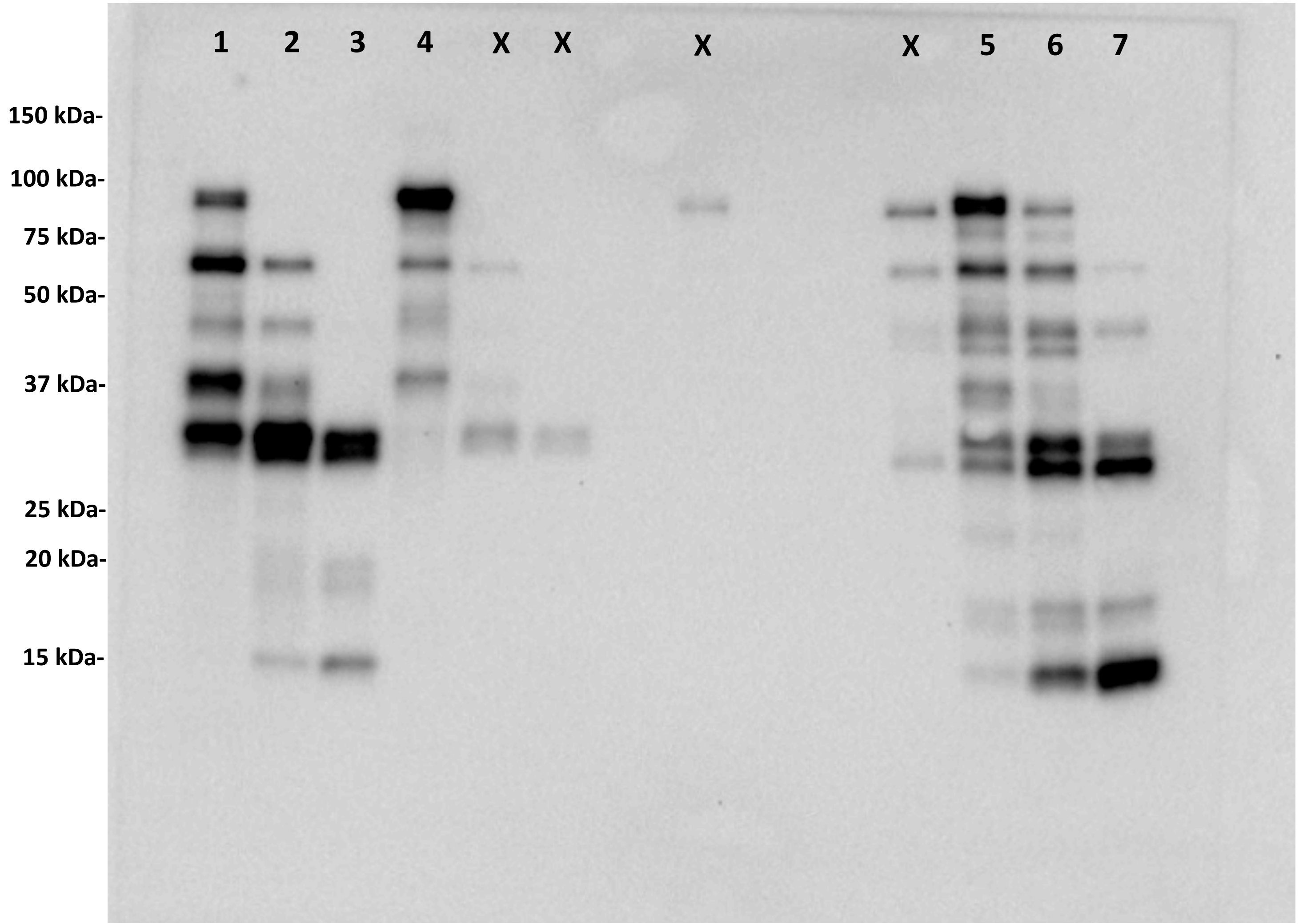

1- Human fecal, 3m
2- Human fecal, 15m
3- Human fecal, 60m
4- Human fecal, 0m
5- Pig intestine, 3m
6- Pig intestine, 15m
7- Pig intestine, 60m

Direction of sample loading: 1 → 7

Image acquisition - ChemiDoc (Bio-Rad)

Fig. 2

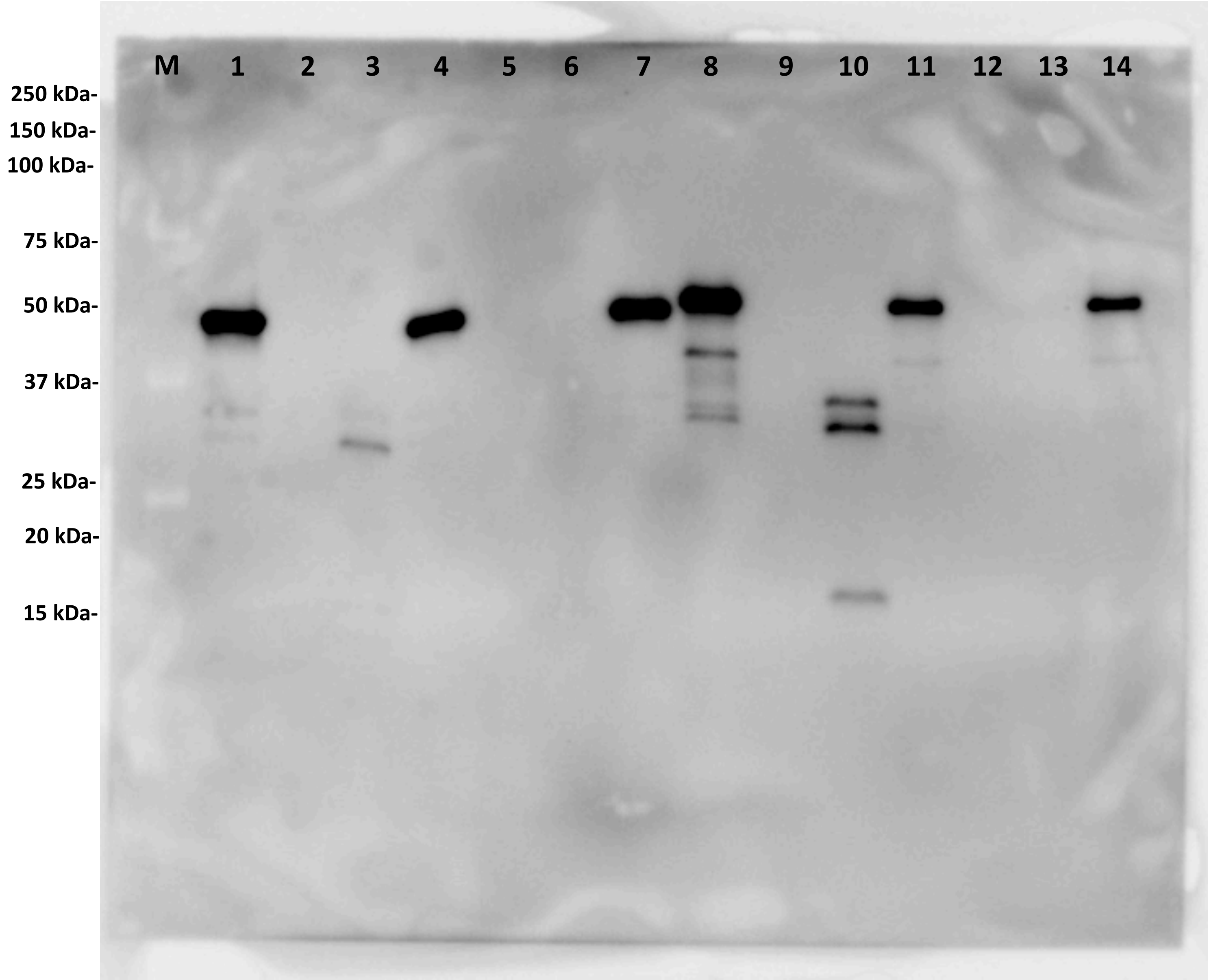

M- molecular weight standard
1- LC/A enzyme inhibitor, PBS, 0 min
2- LC/A enzyme inhibitor, Human fecal, 10 min
3- LC/A enzyme inhibitor, Pig intestine, 10 min
4- LC/A enzyme inhibitor, PBS, 10 min
5- LC/A enzyme inhibitor, Human fecal, 60 min
6- LC/A enzyme inhibitor, Pig intestine, 60 min
7- LC/A enzyme inhibitor, PBS, 60 min
8- Negative control, PBS, 0 min
9- Negative control, Human fecal, 10 min
10- Negative control, Pig intestine, 10 min
11- Negative control, PBS, 10 min
12- Negative control, Human fecal, 60 min
13- Negative control, Pig intestine, 60 min
14- Negative control, PBS, 60 min

Direction of sample loading: M → 14

Image acquisition - ChemiDoc (Bio-Rad)

Fig. S1

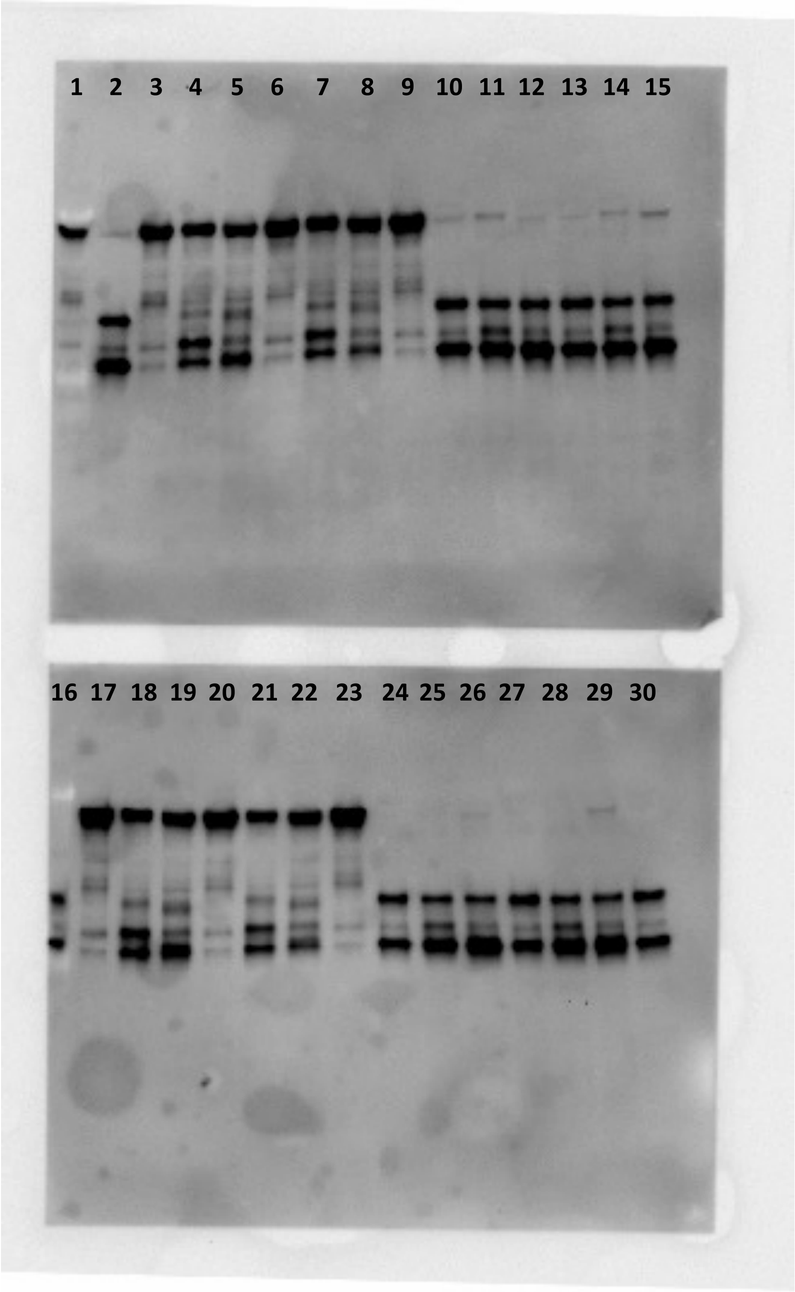

1- No LC/A, 60 m
2- 15 min LC/A incubation, LC/A inhibitor, PBS
3- 15 min LC/A incubation, LC/A inhibitor, PBS 0m
4- 15 min LC/A incubation, LC/A inhibitor, hFecal 10m
5- 15 min LC/A incubation, LC/A inhibitor, pCecum 10m
6- 15 min LC/A incubation, LC/A inhibitor, PBS 10m
7- 15 min LC/A incubation, LC/A inhibitor, hFecal 60m
8- 15 min LC/A incubation, LC/A inhibitor, pCecum 60m
9- 15 min LC/A incubation, LC/A inhibitor, PBS 60m
10- 15 min LC/A incubation, Negative control, PBS 0m
11- 15 min LC/A incubation, Negative control, hFecal 10m
12- 15 min LC/A incubation, Negative control, pCecum 10m
13- 15 min LC/A incubation, Negative control, PBS 10m
14- 15 min LC/A incubation, Negative control, hFecal 60m
15- 15 min LC/A incubation, Negative control, pCecum 60m
16- 60 min LC/A incubation, LC/A inhibitor, PBS
17- 60 min LC/A incubation, LC/A inhibitor, PBS 0m
18- 60 min LC/A incubation, LC/A inhibitor, hFecal 10m
19- 60 min LC/A incubation, LC/A inhibitor, pCecum 10m
20- 60 min LC/A incubation, LC/A inhibitor, PBS 10m
21- 60 min LC/A incubation, LC/A inhibitor, hFecal 60m
22- 60 min LC/A incubation, LC/A inhibitor, pCecum 60m
23- 60 min LC/A incubation, LC/A inhibitor, PBS 60m
24- 60 min LC/A incubation, Negative control, PBS 0m
25- 60 min LC/A incubation, Negative control, hFecal 10m
26- 60 min LC/A incubation, Negative control, pCecum 10m
27- 60 min LC/A incubation, Negative control, PBS 10m
28- 60 min LC/A incubation, Negative control, hFecal 60m
29- 60 min LC/A incubation, Negative control, pCecum 60m
30- 60 min LC/A incubation, Negative control, PBS 60m

Direction of sample loading : 1 → 15 and 16 → 30
Image acquisition - ChemiDoc (Bio-Rad)

Fig. S2A

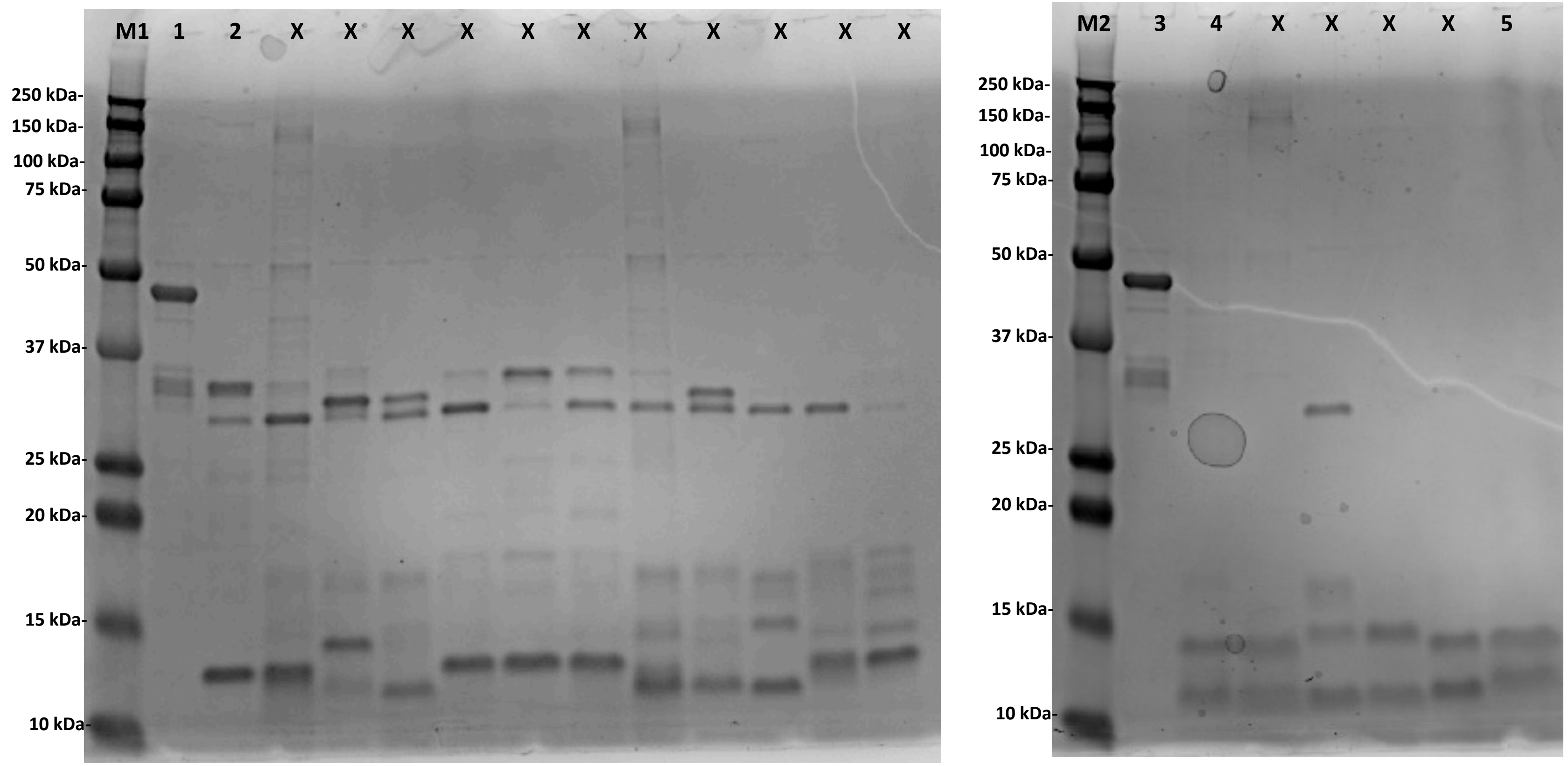

M1- molecular weight standard
1- No incubation, 0m
2- Pig intestine, 60m
M2- molecular weight standard
3- No incubation, 0m
4- Pig intestine, o/n
5- Human fecal, o/n

Direction of sample loading:
M1 → 2 and M2 → 5

Image acquisition - ChemiDoc (Bio-Rad)

Fig. S2B

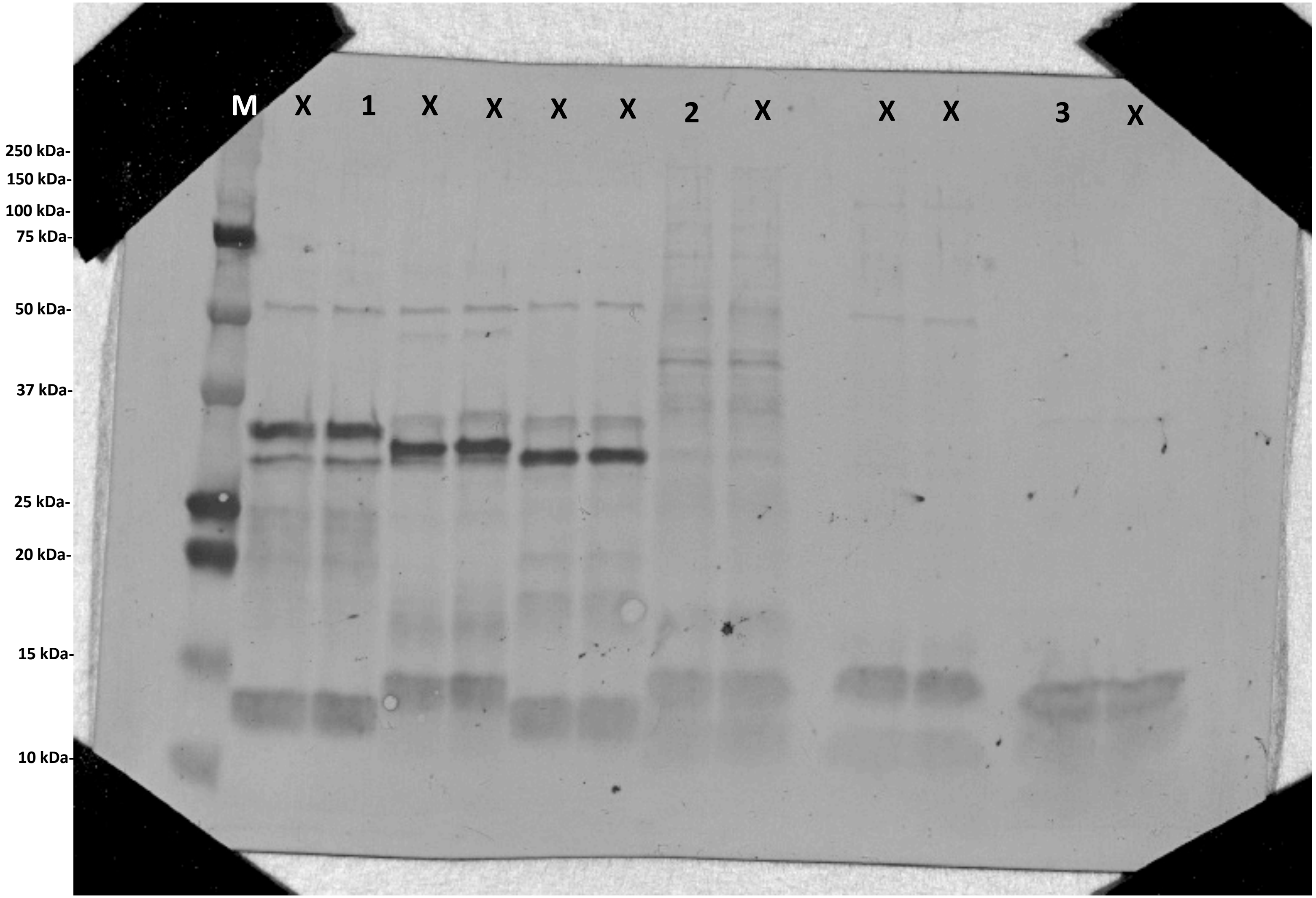

M- molecular weight standards
1- Pig intestine, 60m
2- Pig intestine, o/n
3- Human fecal, o/n

Direction of sample loading: M → 3
Image acquisition - ChemiDoc (Bio-Rad)

Fig. S4A

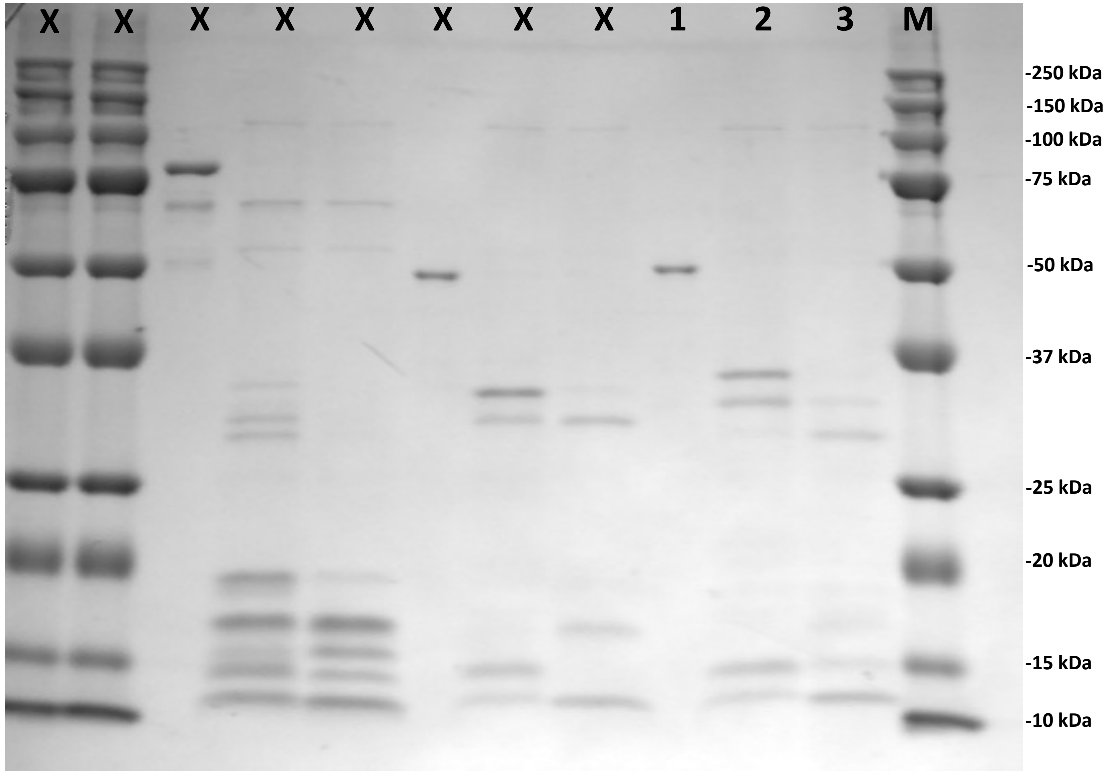

1- No protease, 0
2- Pig intestine, 10m
3- Pig intestine, 60m
M- molecular weight standards

Direction of sample loading: 1 → M
Image acquisition - ChemiDoc (Bio-Rad)

Fig. S4B

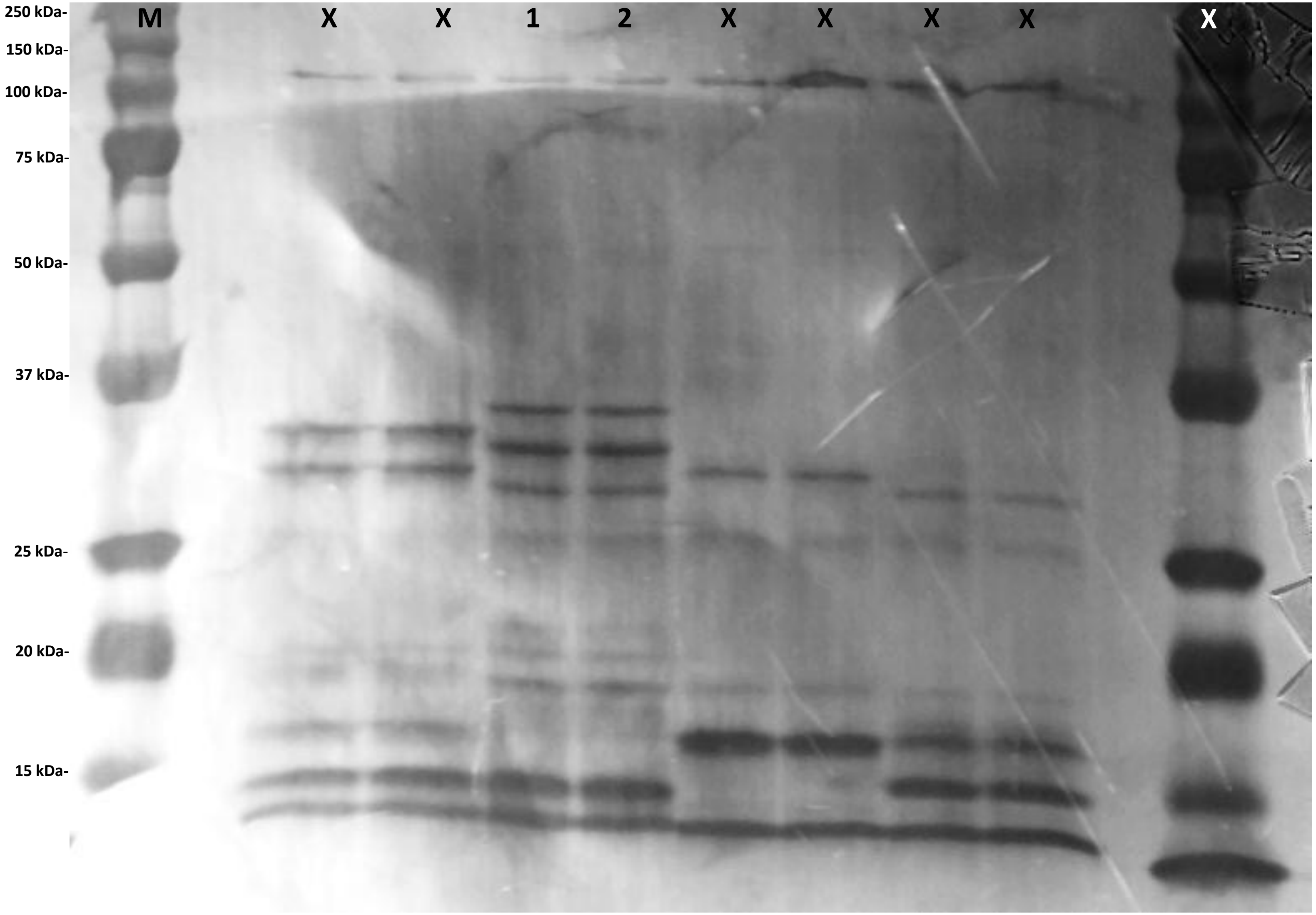

M- molecular weight standards
1- Pig intestine, 60m
2- Pig intestine, 60m

Direction of sample loading: M → 2
Image acquisition - ChemiDoc (Bio-Rad)

Fig. S6

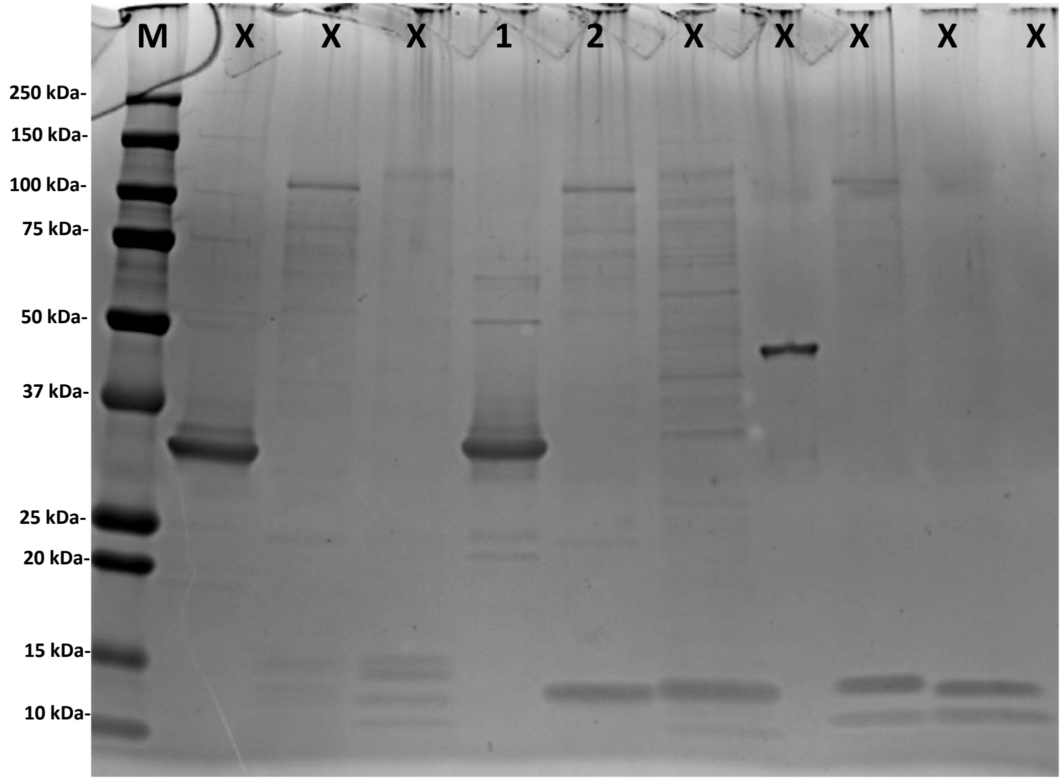

M- molecular weight standards
1- 0
2- Pig intestine, 60m

Direction of sample loading: M → 2
Image acquisition - ChemiDoc (Bio-Rad)

Fig. 4B

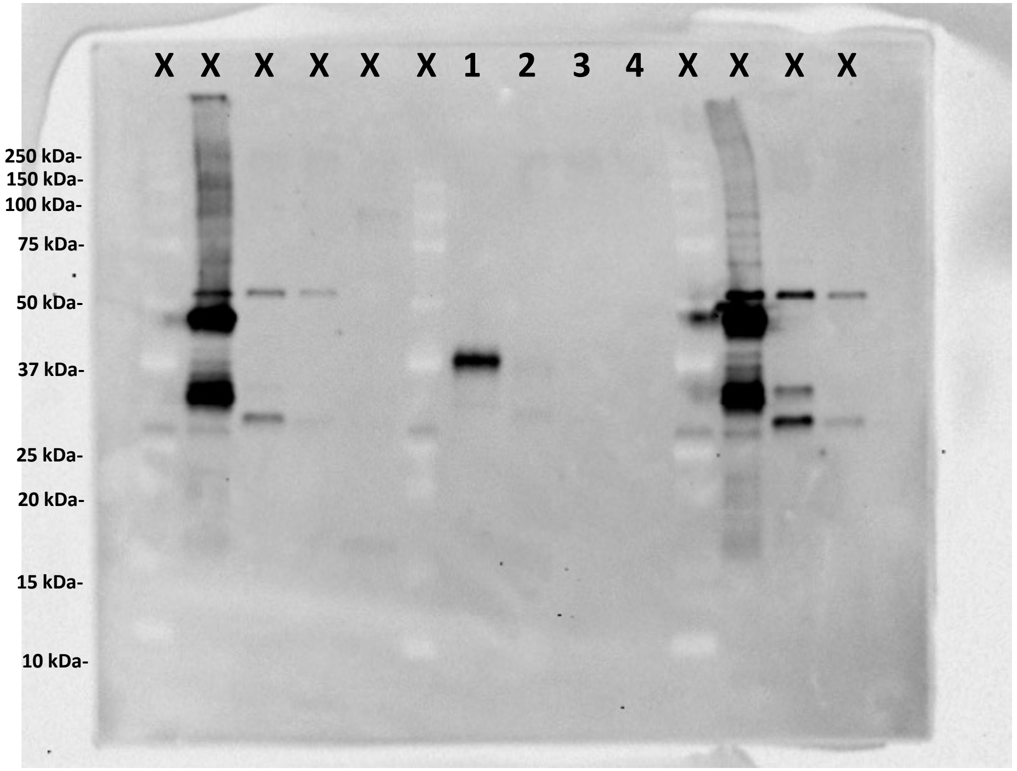

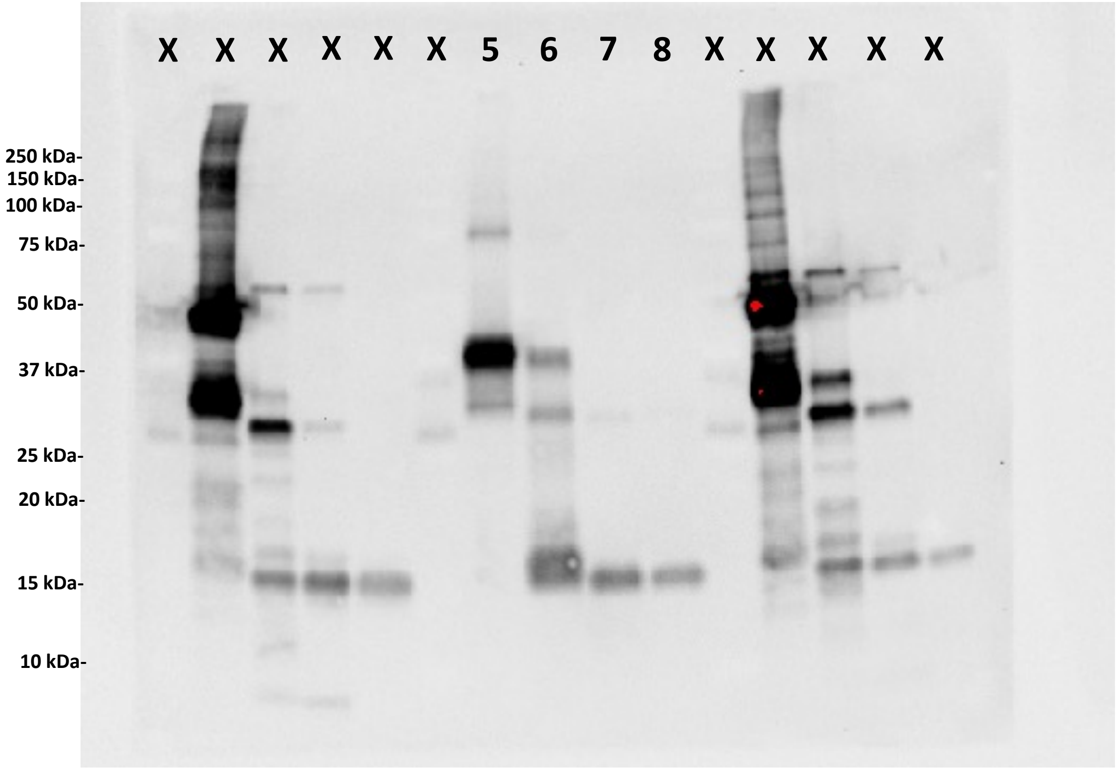

1- Anti-native VHH, 0
2- Anti-native VHH, 30m
3- Anti-native VHH, 3h
4- Anti-native VHH, o/n
5- Anti-denatured VHH, 0
6- Anti-denatured VHH, 30m
7- Anti-denatured VHH, 3h
8- Anti-denatured VHH, o/n

Direction of sample loading: 1 → 4 and 5 → 8
Image acquisition - ChemiDoc (Bio-Rad)

Fig. 5B

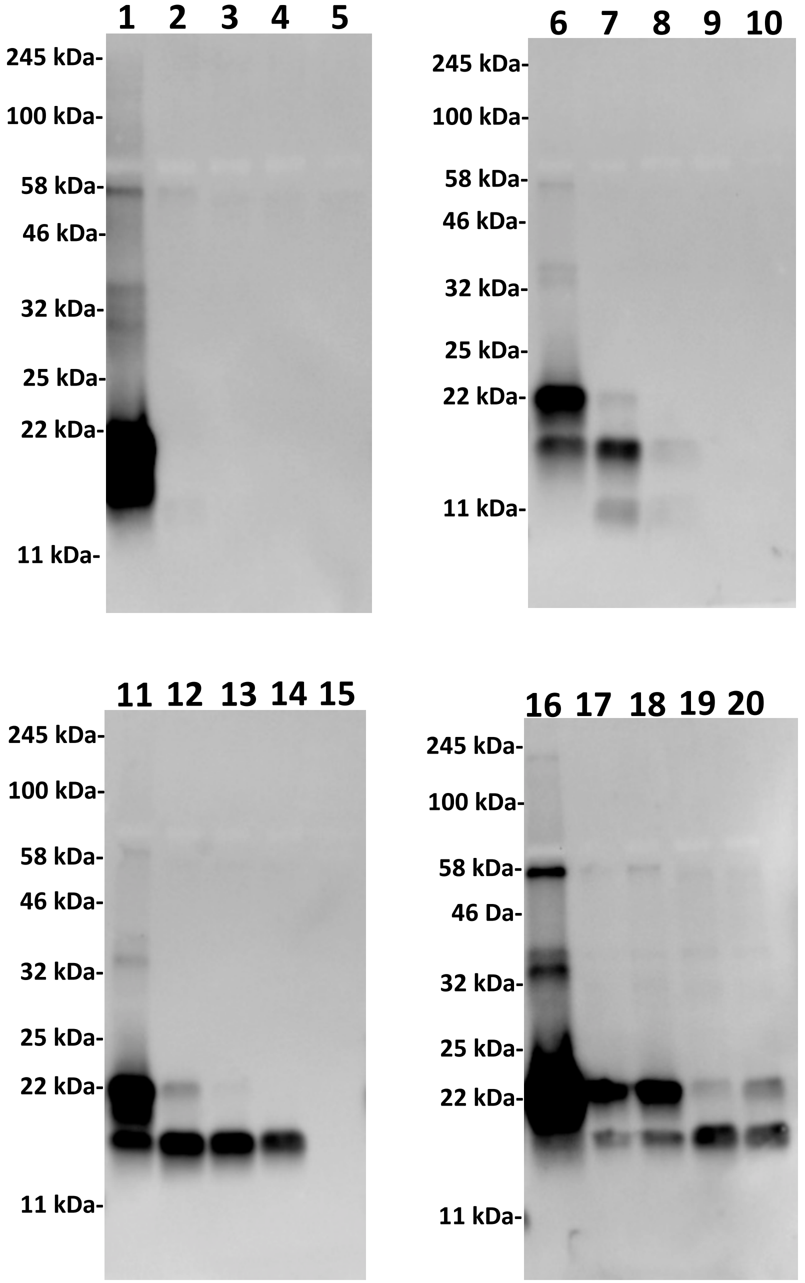

1- JEN-D10, 0
2- JEN-D10, 20m
3- JEN-D10, 1h
4- JEN-D10, 3h
5- JEN-D10, o/n
6- JFG-H6, 0
7- JFG-H6, 20m
8- JFG-H6, 1h
9- JFG-H6, 3h
10- JFG-H6, o/n
11- JGH-G1, 0
12- JGH-G1, 20m
13- JGH-G1, 1h
14- JGH-G1, 3h
15- JGH-G1, o/n
16- JSY-F12, 0
17- JSY-F12, 20m
18- JSY-F12, 1h
19- JSY-F12, 3h
20- JSY-F12, 0/n

Direction of sample loading:
1 → 5, 6 → 10, 11 → 15 and 16 → 20
Image acquisition - ChemiDoc (Bio-Rad)

Fig. 6B

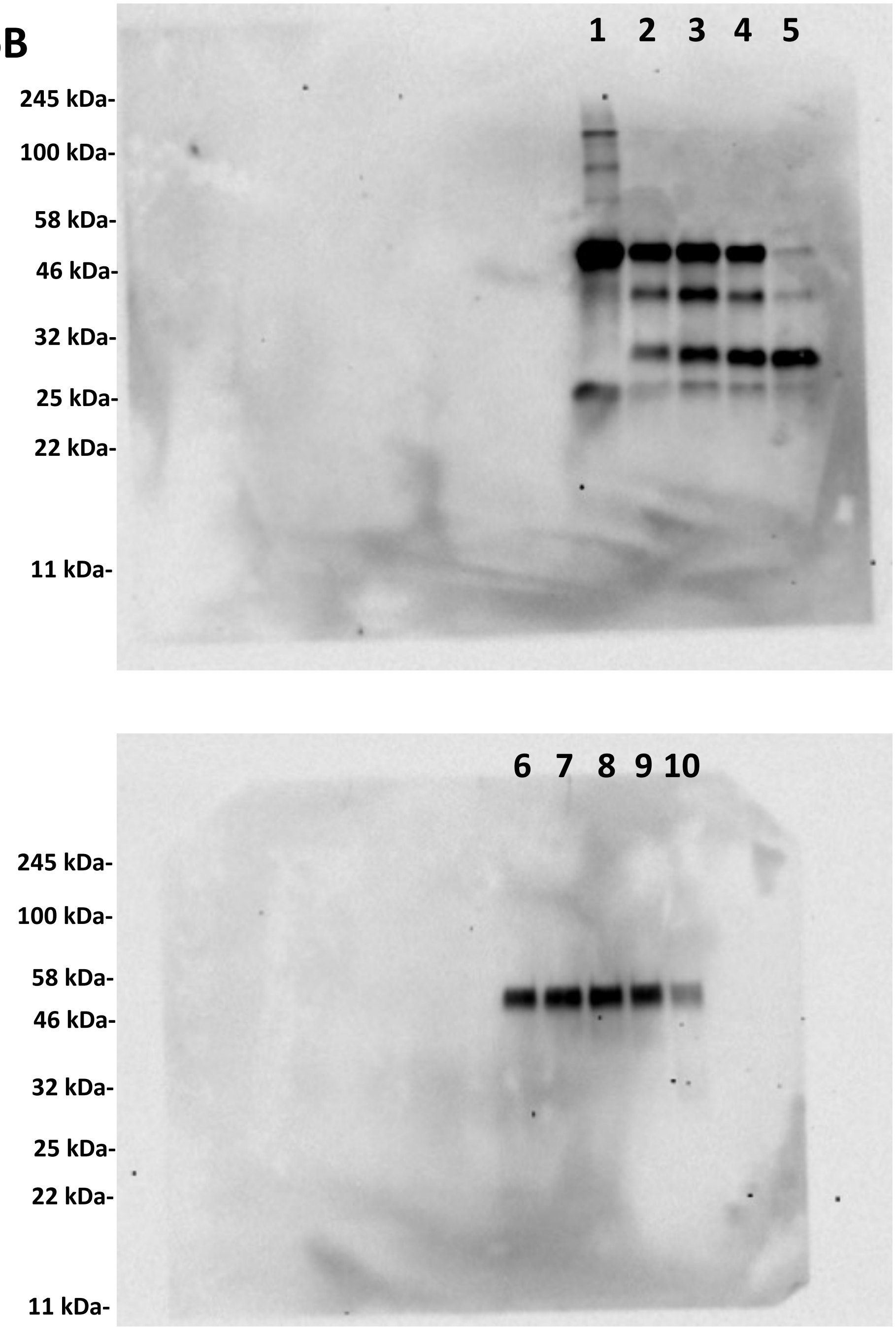

1- Anti-Stx2 5C12 IgG1 mAb, 0
2- Anti-Stx2 5C12 IgG1 mAb, 20m
3- Anti-Stx2 5C12 IgG1 mAb, 1h
4- Anti-Stx2 5C12 IgG1 mAb, 3h
5- Anti-Stx2 5C12 IgG1 mAb, o/n
6- Anti-Stx2 5C12 dIgA2 - pIgR, 0
7- Anti-Stx2 5C12 dIgA2 - pIgR, 20m
8- Anti-Stx2 5C12 dIgA2 - pIgR, 1h
9- Anti-Stx2 5C12 dIgA2 - pIgR, 3h
10- Anti-Stx2 5C12 dIgA2 - pIgR, o/n

Direction of sample loading:
1 → 5 and 6 → 10
Image acquisition - ChemiDoc (Bio-Rad)

Fig. 6B cont.

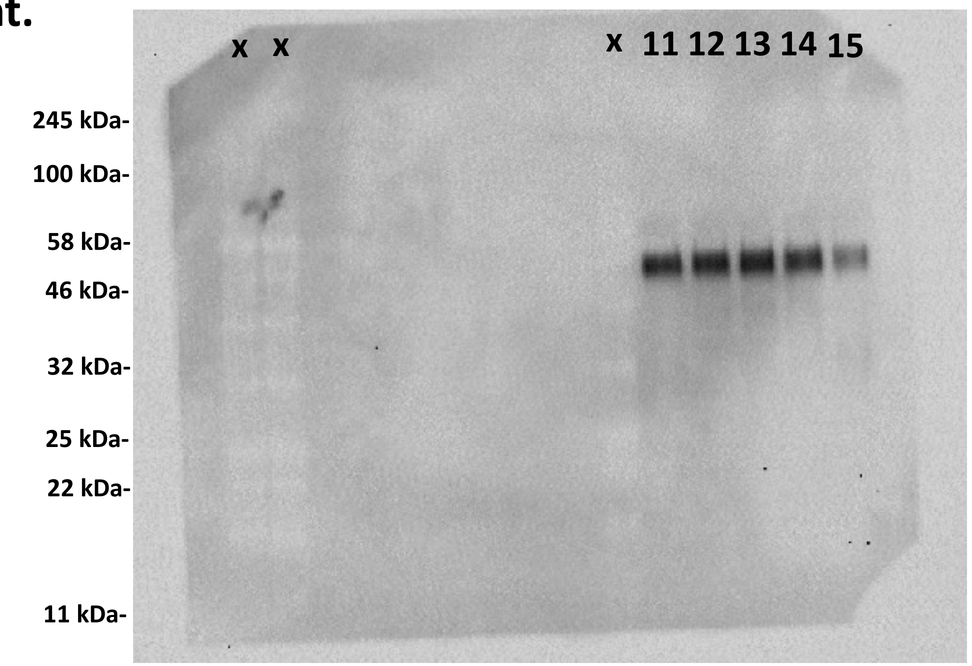

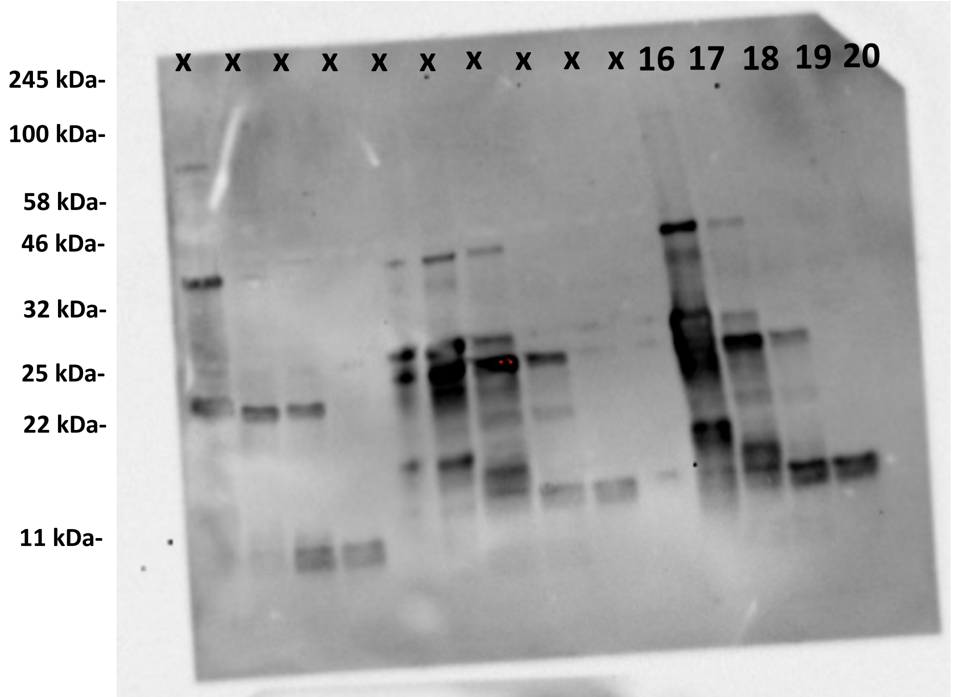

11- Anti-Stx2 5C12 dIgA2 + pIgR, 0
12- Anti-Stx2 5C12 dIgA2 + pIgR, 20m
13- Anti-Stx2 5C12 dIgA2 + pIgR, 1h
14- Anti-Stx2 5C12 dIgA2 + pIgR, 3h
15- Anti-Stx2 5C12 dIgA2 + pIgR, o/n
16- Anti-Stx2 VHH heterodimer, 0
17- Anti-Stx2 VHH heterodimer, 20m
18- Anti-Stx2 VHH heterodimer, 1h
19- Anti-Stx2 VHH heterodimer, 3h
20- Anti-Stx2 VHH heterodimer, o/n

Direction of sample loading:
11 → 15 and 16 → 20
Image acquisition - ChemiDoc (Bio-Rad)

Fig. 7C

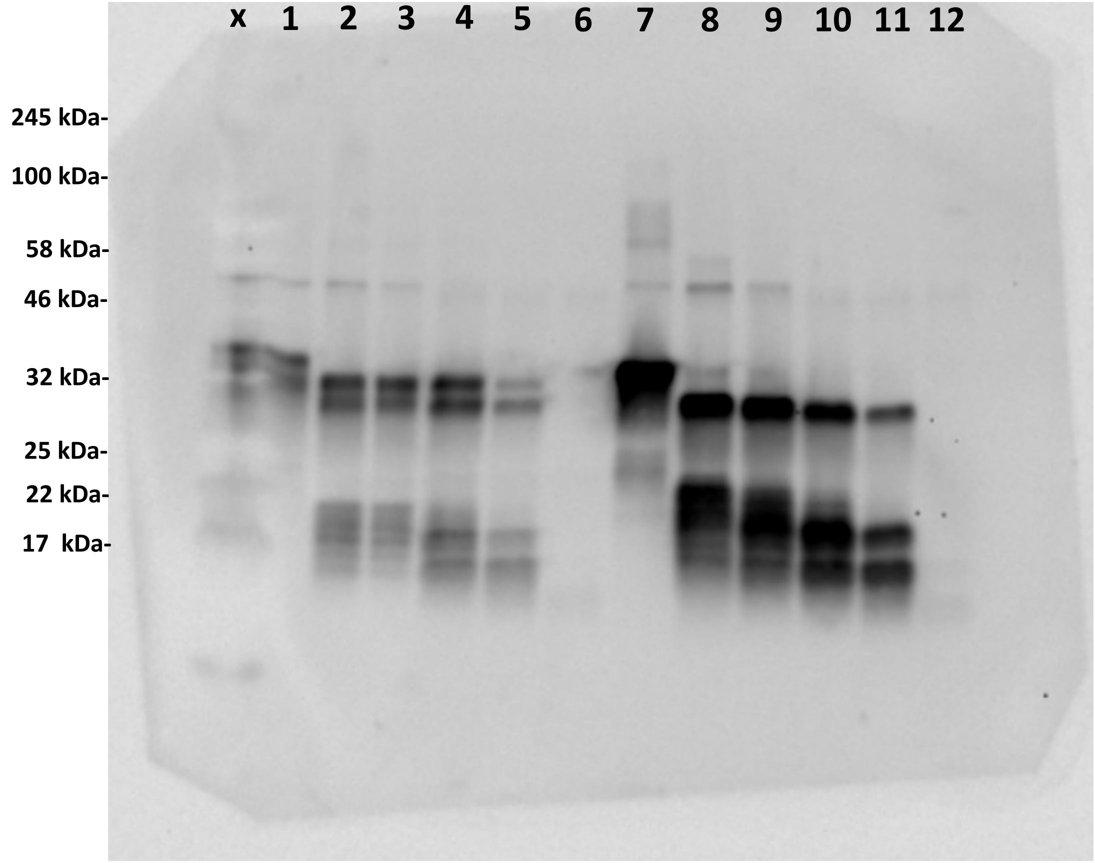

1- 6H/5D/0/E3, 0
2- 6H/5D/0/E3, 30m
3- 6H/5D/0/E3, 1h
4- 6H/5D/0/E3, 2h
5- 6H/5D/0/E3, 4h
6- 6H/5D/0/E3, o/n
7- 6H/PE5/5D/0/E3/PE5, 0
8- 6H/PE5/5D/0/E3/PE5, 30m
9- 6H/PE5/5D/0/E3/PE5, 1h
10- 6H/PE5/5D/0/E3/PE5, 2h
11- 6H/PE5/5D/0/E3/PE5, 4h
12- 6H/PE5/5D/0/E3/PE5, o/n

Direction of sample loading: 1 → 12
Image acquisition - ChemiDoc (Bio-Rad)

Fig. 7 cont.

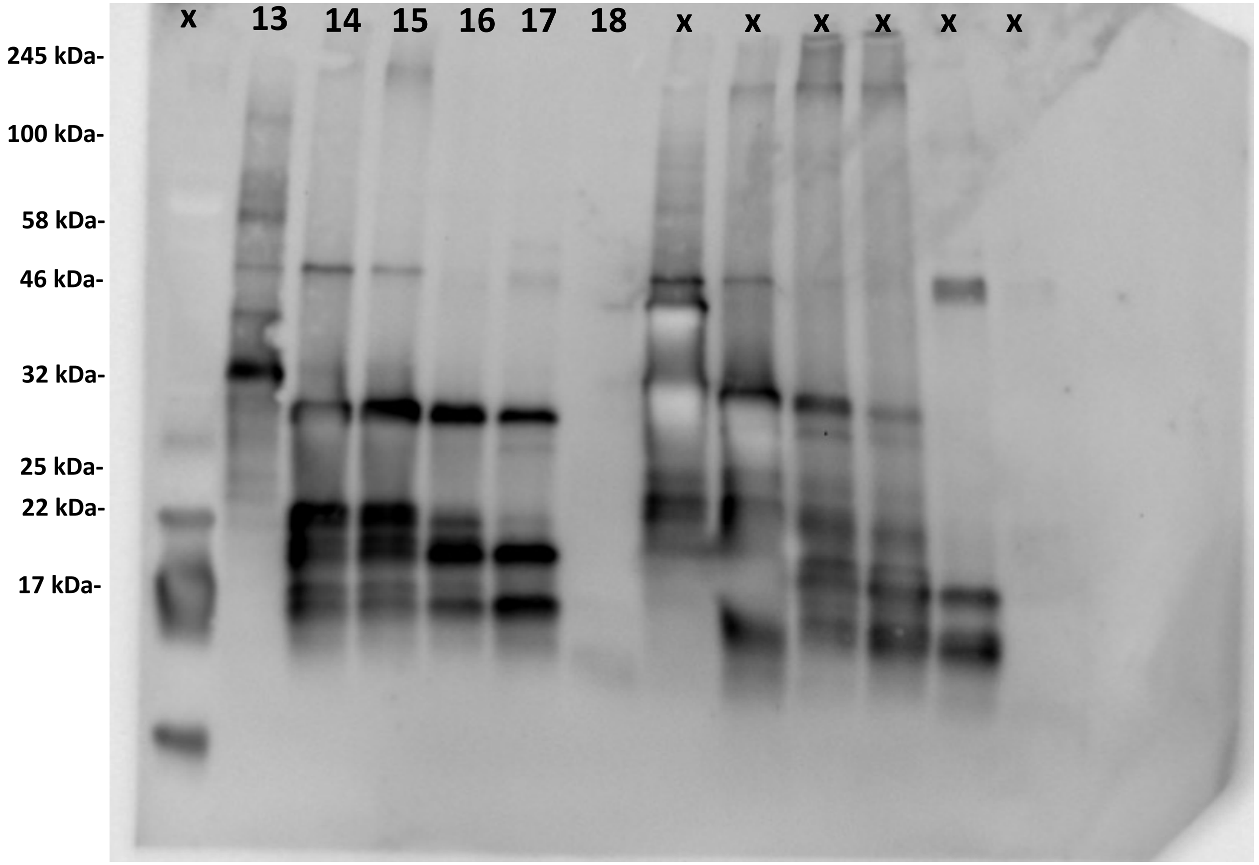

13- 6H/PE5/5D/15G/E3/PE5, 0
14- 6H/PE5/5D/15G/E3/PE5, 30m
15- 6H/PE5/5D/15G/E3/PE5, 1h
16- 6H/PE5/5D/15G/E3/PE5, 2h
17- 6H/PE5/5D/15G/E3/PE5, 4h
18- 6H/PE5/5D/15G/E3/PE5, o/n

Direction of sample loading: 13 → 18
Image acquisition - ChemiDoc (Bio-Rad)

Fig. S8B

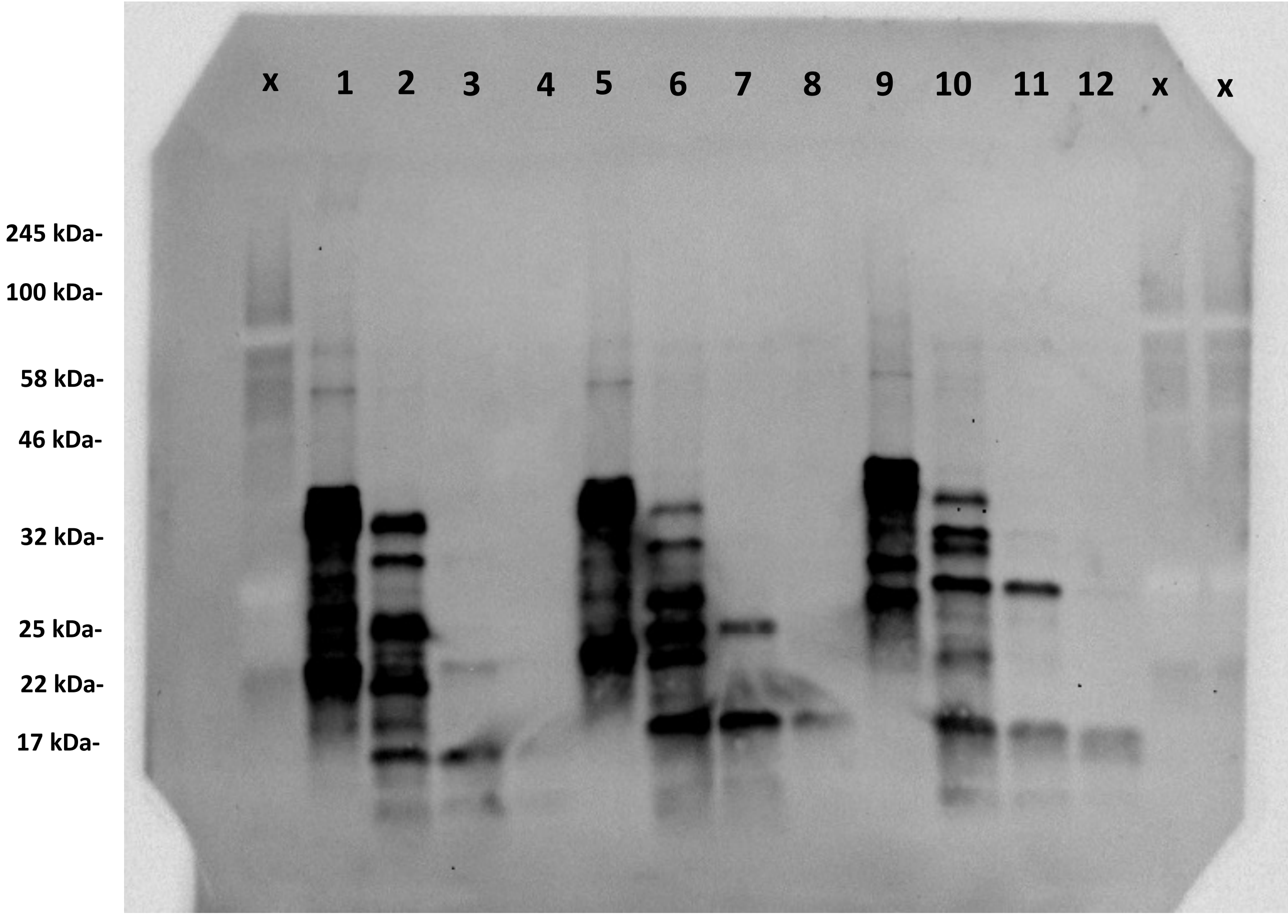

1- 6H/G1/5G/H6, 0
2- 6H/G1/5G/H6, 10m
3- 6H/G1/5G/H6, 30m
4- 6H/G1/5G/H6, 90m
5- 6H/G1/PG3/H6, 0
6- 6H/G1/PG3/H6, 10m
7- 6H/G1/PG3/H6, 30m
8- 6H/G1/PG3/H6, 90m
9- 6H/G1/PE3/H6, 0
10- 6H/G1/PE3/H6, 10m
11- 6H/G1/PE3/H6, 30m
12- 6H/G1/PE3/H6, 90m

Direction of sample loading: 1 → 12
Image acquisition - ChemiDoc (Bio-Rad)
